# A neural network framework for predicting dynamic variations in heterogeneous social networks

**DOI:** 10.1371/journal.pone.0231842

**Published:** 2020-04-27

**Authors:** Mathiarasi Balakrishnan, Geetha T. V.

**Affiliations:** Department of Computer Science and Engineering, Anna University, Chennai, Tamil Nadu, India; Universidad Rey Juan Carlos, SPAIN

## Abstract

Forecasting possible future relationships between people in a network requires a study of the evolution of their links. To capture network dynamics and temporal variations in link strengths between various types of nodes in a network, a dynamic weighted heterogeneous network is to be considered. Link strength prediction in such networks is still an open problem. Moreover, a study of variations in link strengths with respect to time has not yet been explored. The time granularity at which the weights of various links change remains to be delved into. To tackle these problems, we propose a neural network framework to predict dynamic variations in weighted heterogeneous social networks. Our link strength prediction model predicts future relationships between people, along with a measure of the strength of those relationships. The experimental results highlight the fact that link weights and dynamism greatly impact the performance of link strength prediction.

## Introduction

Researchers have tried to understand the fundamental concepts underlying human relationships by analyzing social networks. These studies have opened up a new avenue for predicting future relationships among entities as well. Link prediction has become a hot topic in recent times.

Link prediction is looked upon as a key task in social network analysis and has applications in recommender systems (friend recommendation [1], matrix completion [2] [3]), network inference (finding complete networks based on partial networks), health care (predicting drug-drug interaction) [4] and terror network analysis (finding hidden connections), to name a few. In the case of bibliographic networks, link prediction is used to recommend authors for a review of journals, or as keynote speakers at a conference, or suggest who will collaborate with whom in the future. Likewise, link prediction can help find variations in strengths between author-topic links to discover where the current topic interest of an author lies, learn who the best in a field is, identify reviewers for a paper, choose a keynote speaker for a particular topic, and gauge the affinity between two authors with respect to a venue.

The process of link prediction in static homogeneous networks had its beginnings in the taking of a single snapshot of a network at a particular time for analysis, and predicting future links [5], [6], [7]. The next step included link prediction for evolving networks [8] and weighted networks [9] as well. Actual networks, however, have multiple types of nodes and links that operate in a highly dynamic environment. Researchers have explored link prediction in heterogeneous networks with different types of nodes and links [10]. Earlier work on link prediction in heterogeneous networks overlooked their dynamic characteristics [11]. Recently, weighted but non-dynamic heterogeneous networks have been considered for link prediction [12]. Weighted dynamic heterogeneous networks help capture dynamic variations in link strengths in a complex network. In this paper, we propose a link strength prediction framework in dynamic, weighted heterogeneous networks. Further, we take into account the link information at different time slots, thereby capturing the dynamic evolution of the heterogeneous network.

The major contributions of our proposed work include:

- Link strength prediction in dynamic and weighted heterogeneous networks with reference to a bibliographic network wherein relationship strength prediction between pairs of authors is made on the basis of the paper, topic and venue. Usage of weighted meta-path-based features for learning.
- Link strength prediction for different time granularities, and an analysis of the time granularity at which the weight of a link changes.
- Modification of the kernel initializer for the initialization of weights in the neural network, thereby facilitating a more accurate prediction of link strengths.

The rest of the paper is organized as follows. Section 1 details about related work done in link prediction. Section 2 describes the problem statement which is followed by Sections 3, 4 and 5 which elaborate on the proposed method. Section 6 describes about the ARIMA (Auto Regressive Integrated Moving Average) model and the learning framework used. Section 7 describes the experiments conducted and their results along with the evaluations done. Section 8 is reserved for conclusion and future work.

## 1 Related work

### 1.1 Homogeneous versus heterogeneous networks

**Fig 1:**
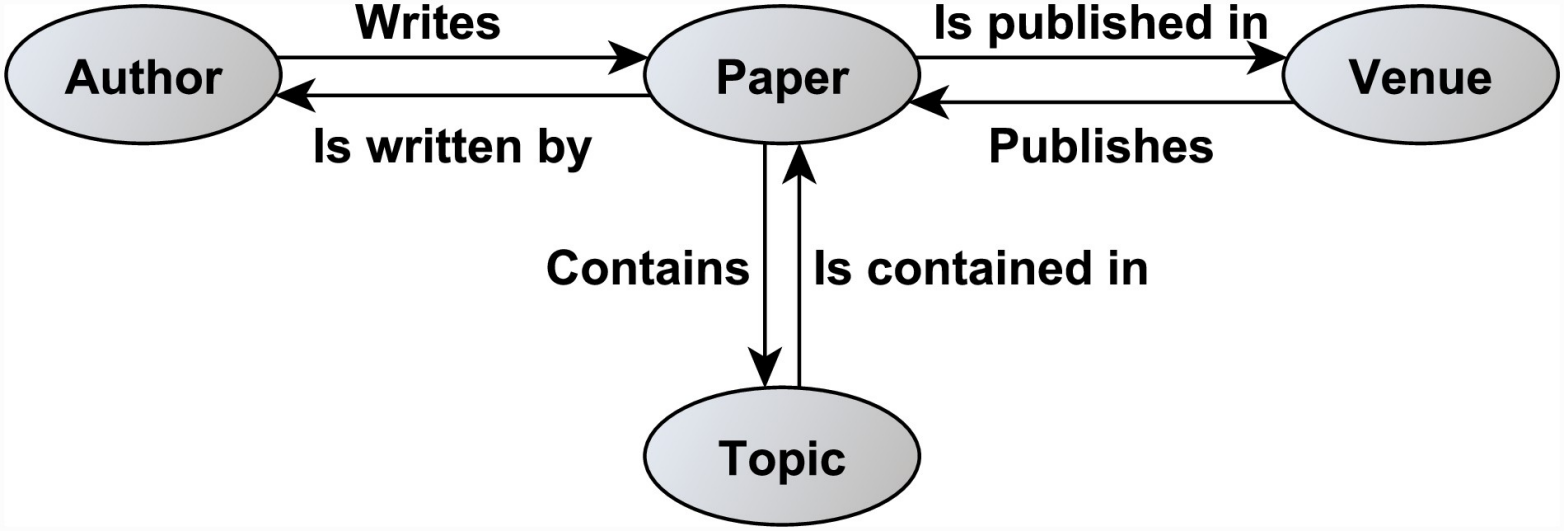
DBLP network schema.

Much work on link prediction has been done in homogeneous networks with the same type of nodes and links [13], [14], [15], [16] but with the heterogeneous aspect of the network largely ignored. Using a homogeneous network facilitates the modeling of a real-world network. For example, a bibliographic network can be modeled as a homogeneous network where the nodes are ‘authors’ and the links are of the type ‘is a co-author of’. The same bibliographic network can be modeled as a heterogeneous network as well, where the nodes belong to multiple types such as authors, papers, venues/conferences and topics. The network schema of such a bibliographic network is seen in Fig 1.

Two approaches can be used to predict links: score-based and learning-based. In the score-based approach, the similarity scores of node pairs that are not connected in the network are computed, following which the scores are ranked in descending order to predict new links. The higher the score for a particular node pair, the greater the probability that the two nodes will connect with each other in the future.

Papadimitriou et al. [17] employed a path-based topological feature that takes the count of all paths of varying lengths as features, while Xu et al [13] used path entropy as a feature, where a large link entropy implies that there is only a small probability for the node pairs to be linked in future. Community relevance [14] is another path-based topological feature used for link prediction. Neighborhood topological features such as Common Neighbors, and event-based topological features [15] were also used for link prediction in homogeneous networks. Tsugawa and Kito [18], have predicted links by analyzing retweets that include Retweet views and Retweet posts. Their network is homogeneous, where nodes are considered as users. Node pairs are ranked in descending order of their link prediction scores, following which the Borda score is used for rank aggregation. Their work predicts future relationships, using static snapshots of the network. As is evident, score-based methods are mostly applied to homogeneous networks.

In the learning-based link prediction approach, which treats the link prediction problem as a binary classification task, machine learning models are used to resolve it. Li and Chen [19] used a kernel-based recommendation approach with node-based features. A kernel is designed on user-item pairs based on their context, structure and features, and an SVM algorithm used to predict the links. Ahmed et al [20] used topological features and an ensemble of classifiers such as the Rotation Forest, AdaBoost, Dagging and Random Forest to predict links on Twitter. In both the papers, only static networks were considered. Node-based features work well only in small-sized networks and multi-nodal relationships cannot be identified in these cases.

An unsupervised technique called spectral clustering was used by Symeonidis and Mantas [21], where the top-k eigenvectors and the corresponding eigenvalues of the normalized Laplacian matrix are computed, and approximately equal elements in the selected eigen vectors are clustered using the K-Means. Similarity indexes are computed for all the node pairs, based on the distance of each node from the nearest cluster centroid. The top-ranked ones are recommended as links to a particular node. Here again, only static homogeneous networks were considered, ignoring the dynamic changes in the network.

Recently, Graph Neural Network has gained a lot of importance and attention. A Graph Neural Network is a special kind of Neural Network which directly acts on a graph. Zhang and Chen [16] proposed a new framework called SEAL where the heuristic that explains link formations is learned using a graph neural network. For a given link in a homogeneous network, the subgraphs surrounding the link are given as input, and output is the likelihood of the existence of the link. Another significant work is based on variational auto-encoder [22]. A graph convolutional network is used to demonstrate this model, which learns the latent representations for undirected graphs. Using this proposed auto-encoder, embeddings of an unweighted graph are arrived at, based on which future links are predicted.

Davis et al. [23] employed a weighted extension of neighborhood-based topological features which included Preferential Attachment and Common Neighbors to predict links in a heterogeneous network. They used a supervised training algorithm, bagging, in tandem with random forests, within each bag. Hadi-Shakibian et al. [24] used meta-path-based topological features where several training sets were created for each meta-path relation. The least-squares twin-support-vector machine algorithm was applied on each set of training data, and majority voting used to obtain the final link prediction. A static heterogeneous network was considered for their work. Recent research has focused on link prediction in heterogeneous networks using supervised ranking on the basis of meta-path-based features [25]. The temporal aspect, however, has not been touched on. When considering a static snapshot of the network, the characteristics of the network (features) at that point of time are only used for the computation of future links. But the activities of people keep changing gradually. In case of Twitter/Facebook, people keep posting status updates; in bibliographic networks, people publish more papers and collaborate with new authors in varied topics, probably in a new venue. These changes are reflected in the network and further in its characteristics/features. So, it is important to consider dynamism as these state changes over time help in figuring out the formation of new links with the progress of time thereby increasing the accuracy of prediction.

### 1.2 Link strength prediction in weighted networks

Link strength plays a vital role in the analysis of social relationships [26]. Khosravi et al. [27] proposed a relationship strength prediction method where a matrix factorization model was used to predict the strength of an existing link. Here, too, only a static network was considered. Moreover, link strengths are provided by users in the form of a matrix and are not calculated on the basis of the network structure. Yang and Yang [28] detected drug-drug interaction signals based on the Weighted Path Count feature in a heterogeneous health care network, while overlooking the dynamic aspect of the network. Kahanda and Neville [29] predicted the strength of a relationship using supervised learning in a homogeneous network. Temporal changes in the network were not considered for the study.

### 1.3 Link prediction in dynamic networks

Networks evolve continuously. Taking a single snapshot of a network at a particular time results in a failure to capture crucial changes occurring in the network state at different times. Considering the dynamic aspect of the network [8], the network state at different times can be captured, which helps in an analysis of the variations in relationships with respect to time.

When considering the dynamic aspect of the network, a series of network snapshots at regular time intervals, say, *G*_1_, *G*_2_…*G*_*t*_, is to be collected and the likely link state at the time t+1, *G*_*t*+1_ predicted. In this scenario, new edges may be added and existing ones deleted, while new nodes may come into existence and existent nodes vanish.

Ozcan and Oguducu [30] used global and local similarity measures in heterogeneous networks for multivariate time series link prediction. The network they considered has multi-typed links with only a single type of node.

Li et al. [31] studied link prediction in dynamic homogeneous networks using neighborhood-based features that capture the neighbors’ influence. This was done using a deep learning framework comprising the Conditional Temporal Restricted Boltzmann Machine (ctRBM) Neighbor influence clustering algorithm to reduce computational complexity and highlight the fact that group behavior is much more stable and predictable than individual behavior. Gunes et al. [32] used neighborhood-based topological features such as the Common Neighbors, Adamic Adar, Jaccard co-efficient and preferential attachment to predict links in both weighted and unweighted homogeneous networks. They used a time series forecasting model called ARIMA for future node similarity scores. Sun et al., [33] in their work, examined the prospect of when a relationship was likely to occur in a heterogeneous network, though they did not consider the strength of the links.

Clusters using structural similarity and nodal attributes, employing an incremental clustering algorithm, were created by Aggarwal et al., [34] where the frequencies of attribute pairs and related frequencies are considered in a dynamic heterogeneous network. Decay-based frequencies were tracked over time to determine the prediction score between a pair of nodes.

The analysis above shows that there are still some open research issues:

- To the best of our knowledge, relationship strength prediction in dynamic weighted heterogeneous networks (with multi-typed nodes as well as links) has not been undertaken yet.
- Variations in relationship strength with respect to time have not been considered.
- Changes in relationships in different granularities of time have not been analyzed.

## 2 Problem definition

Let G be a weighted heterogeneous network, represented by G(V,E,w), where V is the set of all types of nodes, E the set of all types of edges, and w the weight of the edges. The node type maps to the function, *ν*: *V* → *N*, and the edge type has a mapping function, *ξ*: *E* → *L*. Each node, *v* ∈ *V*, refers to a particular node type in the node set, *N*: *ν*(*v*)∈*N*, and each link, *e* ∈ *E*, refers to a particular link type in the relationship set, *L*: *ξ*(*e*)∈*L*.

Consider a series of snapshots of such a weighted heterogeneous network, *G*_1_, *G*_2_…*G*_*t*_, taken at time slots *t*_1_, *t*_2_…*t*_*t*_. We propose a link strength prediction method which predicts the strengths of the links of the network, *G*_*t*+1_. The proposed link strength prediction method uses a combination of the ARIMA (Autoregressive Integrated Moving Average) time-series forecasting, and a neural network framework to arrive at the weighted heterogeneous network at time *t* + 1.

## 3 Basic concepts

### 3.1 Meta-path

A meta-path is a path comprising different kinds of relationships among nodes of different types. It can be obtained by traversing the network schema of the network under consideration.

For example, in a bibliographic network, the following are some of the possible meta-paths:

1. Author→Paper→Author—This meta-path refers to the co-author relationship.
2. Author→Paper→Topic→Paper→Author—This meta-path refers to the common topic which is shared by two research papers.
3. Author→Paper→Venue→Paper→Author—This meta-path connects the authors who publish their papers in the same venue.

These paths can be obtained from the DBLP network schema shown in Fig 1.

### 3.2 Constructing a weighted network

The primary reason for considering a weighted network is that not all edges have equal strength or importance. Moreover, link weights play a major role in link formation and dissolution. There are several ways in which a network can be weighed and in this paper, we weigh it based on link leverage or importance.

In association rule mining, given an association rule of the form I→J, *S*_*ij*_ refers to Support(I→J), which is the frequency of occurrence of the association. *S*_*i*_ refers to Support(I), which is the frequency of occurrence of I, and *S*_*j*_ refers to Support(J), which is the frequency of the occurrence of J.

The leverage of the association, I→J, is given by Eq 1, where S refers to Support.

$$\begin{array}{c} \text{Link}\;\text{Leverage(I}\to \text{J)}=S_{ij}-S_{i}*S_{j} \end{array}$$ (1)

This can be extended to compute the importance of a link, *L*_*ab*_, between two nodes, *N*_*a*_ and *N*_*b*_, as specified in [28], where a and b refer to any two entities (authors/papers/topics/venues). The link importance is computed by Eq 2.

$$\begin{array}{c} \text{Link}\;\text{Importance}=S\left(L_{ab}\right)-S\left(N_{a}\right)*S\left(N_{b}\right) \end{array}$$ (2)

where Support(*L*_*ab*_) or S(*L*_*ab*_) is given by Eq 3. In this scenario, we consider every research paper as a transaction and the database is considered the set of all such transactions, i.e., research papers.

$$\begin{array}{c} S\left(L_{ab}\right)=\frac{\text{Link}\;\text{Frequency}(L_{ab})}{n} \end{array}$$ (3)

In Eq 3, *L*_*ab*_ denotes the link between nodes a and b that are specified as *N*_*a*_ and *N*_*b*_, and ‘n’ is the number of transactions in the interval of the time period considered. Link Frequency(*L*_*ab*_) is defined as the frequency of occurrence of the link *L*_*ab*_. The support values for the nodes are given by Eqs 4 and 5. In these equations, Node Frequency(*N*_*a*_) and Node Frequency(*N*_*b*_) refer to the frequency of occurrence of the nodes, *N*_*a*_ and *N*_*b*_ respectively.

$$\begin{array}{c} S\left(N_{a}\right)=\frac{NodeFrequency(N_{a})}{n} \end{array}$$ (4)

$$\begin{array}{c} S\left(N_{b}\right)=\frac{NodeFrequency(N_{b})}{n} \end{array}$$ (5)

### 3.3 Meta-path-based features for a weighted network

Most commonly, when considering link formation between two nodes, their structural similarity plays a significant role. Features in homogeneous networks that define the structural similarity between two nodes include the Common Neighbors, Jaccard co-efficient, Adamic Adar, and preferential attachment. However, for heterogeneous networks with their multiple types of nodes and links, it is impossible to directly define these features. Hence we consider meta-path-based features for such a network.

Moreover, weights help differentiate the strengths of links between different pairs of authors and, likewise, help in a better understanding of the evolution of link strengths across different time periods. Hence, we propose four weighted meta-path-based features for link strength prediction, namely, Weighted-Path-Count, Normalized-Weighted-Path-Count, Weighted-Activity-Ratio and Weighted-Symmetric-Activity-Ratio.

The Weighted-Path-Count is the weighted extension of Common Neighbors feature of homogeneous networks, which is the count of total number of paths following a particular meta-path relation, as in, for example, Author→Paper→Author in a bibliographic network. The Weighted-Path-Count (*WPC*) is given by Eq 6.

$$\begin{array}{c} WPC_{R}=\frac{1}{Length}\sum_{Path}\sum_{i=1}^{Length}Weight\left(n_{i},n_{i+1}\right) \end{array}$$ (6)

*Length* denotes the length of the meta-path and *R*, the meta-path relation. In the equation, *n*_*i*_ and *n*_*i*+1_ represent the nodes that are connected to each other following the meta-path relation R, and *Weight*(*n*_*i*_, *n*_*i*+1_) represents the ‘Link importance’ that was computed for the link between the nodes *n*_*i*_ and *n*_*i*+1_ using Eq 2 (which acts as the weight of the link). For example, consider a meta-path relation *A*_1_ − *V* − *A*_2_ (Two authors who are connected by many venues). The weights of all the links between *A*_1_ − *V* and *V* − *A*_2_ are summed up and divided by the length of the meta-path relation A-V-A, which is 2. The next feature is the Normalized-Weighted-Path-Count (*NWPC*), given by Eq 7.

$$\begin{array}{c} NWPC_{R}=\frac{WPC_{R}\left(a_{i},a_{j}\right)+WPC_{R^{-1}}\left(a_{j},a_{i}\right)}{WPC_{R}\left(a_{i},.\right)+WPC_{R^{-1}}\left(.,a_{j}\right)} \end{array}$$ (7)

*WPC*_*R*_ is the weighted path count for the meta-path relation R, *WPC*_*R*^−1^_ is the weighted path count of the inverse of the relation R, *WPC*_*R*_(*a*_*i*_,.) is the sum of all the weighted path counts for the relation R starting with node *a*_*i*_, and *WPC*_*R*^−1^_(., *a*_*j*_) is the sum of all the weighted path counts for the inverse of the relation, R ending with node *a*_*j*_.

Similarly, Weighted-Activity-Ratio (*WAR*) (refer to Eq 8) gives the activity ratio of author pairs in a heterogeneous network, and is based on the prop flow, defined in [35].

$$\begin{array}{c} WAR_{R}=\frac{WPC_{R}\left(a_{i},a_{j}\right)}{WPC_{R}\left(a_{i},.\right)} \end{array}$$ (8)

In Eq 8, *WPC*_*R*_(*a*_*i*_,.) is the sum of all the weighted path counts for the relation R, starting with the node, *a*_*i*_, and ending at any node.

The Weighted-Symmetric-Activity-Ratio (*WSAR*) feature of the heterogeneous network takes into consideration the activity ratio of author pairs from two directions along the meta-path relation, which is provided by Eq 9.

$$\begin{array}{c} WSAR_{R}=WAR_{R}\left(a_{i},a_{j}\right)+WAR_{R^{-1}}\left(a_{j},a_{i}\right) \end{array}$$ (9)

In Eq 9, *WAR*_*R*^−1^_ refers to the Weighted-Activity-Ratio feature for the inverse of the meta-path relation, R.

## 4 Autoregressive Integrated Moving Average (ARIMA)

Autoregressive Integrated Moving Average or ARIMA, is a time series forecasting model which is used for predicting future points in the series. The autoregressive (AR) part regresses the variable on its past values, while the moving average (MA) part models the error term. The integrated (I) part indicates that the data values are replaced with the difference between the current and previous values. The model uses three variables: *p*, which specifies the number of autoregressive terms; *d*, which specifies the number of non-seasonal differences needed to introduce stationarity in the time series data, and *q*, which is the number of lagged forecast errors in the predicted value and specified as ARIMA (*p*,*d*,*q*).

There are various forecasting models [36] used for predicting future values. The first one is to simply use the feature values at the current time period (from *G*_*t*_) to predict the future links. This is like considering a static snapshot of the network and hence, does not serve our purpose. The next forecasting technique is to take the average of the feature values taken from the first time slot to time slot t. However, this method does not give the correct prediction. For example, suppose the feature values are in descending order (and diminish with time), then the feature value at a future time will be the smallest of all the values previously seen. Averaging does not capture this. ARIMA, on the other hand, can capture complex relationships as the error terms and observations of lagged terms are taken into consideration. ARIMA model relies on auto-regression, which is the process of regressing a variable on its past values. Autocorrelations eventually decay which gives the estimate of the degree to which white noise characterizes a data series. The process of finding the coefficients of the model is by recursively calculating them until a proper fit with the actual data is obtained.

## 5 Link strength prediction model

The objective of this work is to predict relationship strength at a future time interval, based on the strengths of the relationships at past and current time intervals. To achieve this objective, network snapshots *G*_1_, *G*_2_, *G*_3_…*G*_*t*_ are collected at regular time intervals, right up to the current time, *t*. Each of these graphs is weighed according to the procedure elaborated in Section 3.2 for every meta-path relation. The weights assigned to the edges vary and are based on link importance, otherwise known as link leverage. Following this, weighted meta-path-based features are extracted for each of the graphs *G*_1_, *G*_2_…*G*_*t*_, based on the formulae given in Section 3.3. These features are now fed into the ARIMA model to forecast the weighted feature values at a future time interval, *t* + 1.

**Fig 2:**
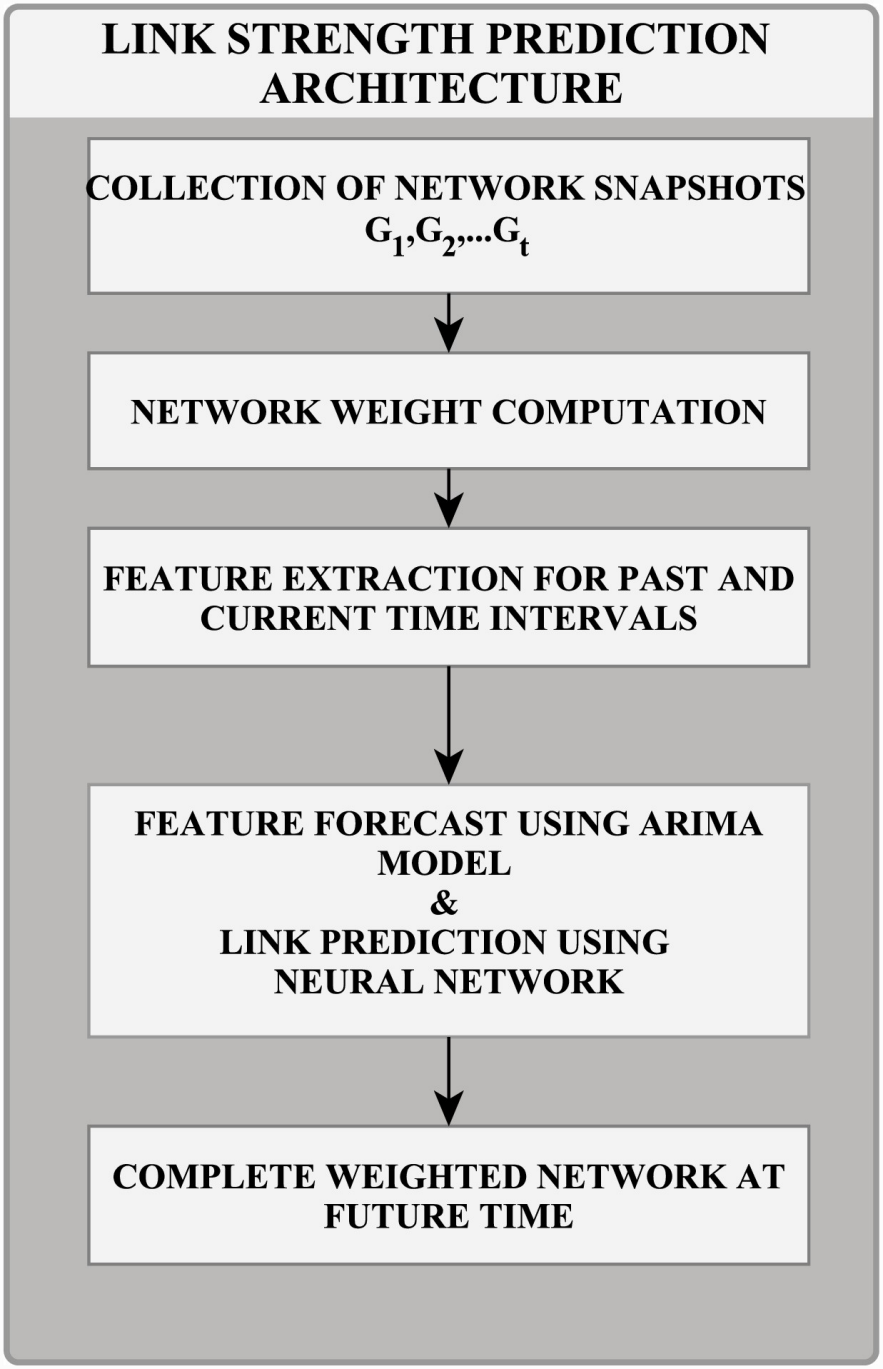
Architectural framework.

The future values of these features are fed into a neural network that predicts the relationship strength between two nodes at time *t* + 1. The same procedure was repeated for every meta-path relation. Fig 2 shows the architectural framework of our proposed work.

## 6 Neural network framework for link strength prediction

The ARIMA model was used to forecast feature values for a future period in time. Given a series of time-sensitive data, this model predicts the future value of the data. This is based on the past and current values, as well as on modeling the error as a linear combination of errors that occurred in those values.

The ARIMA model provides the feature values for the future time period, t+1, which are given as input features for the neural network which, in turn, predicts the link strengths for author pairs at a future period in time.

**Fig 3:**
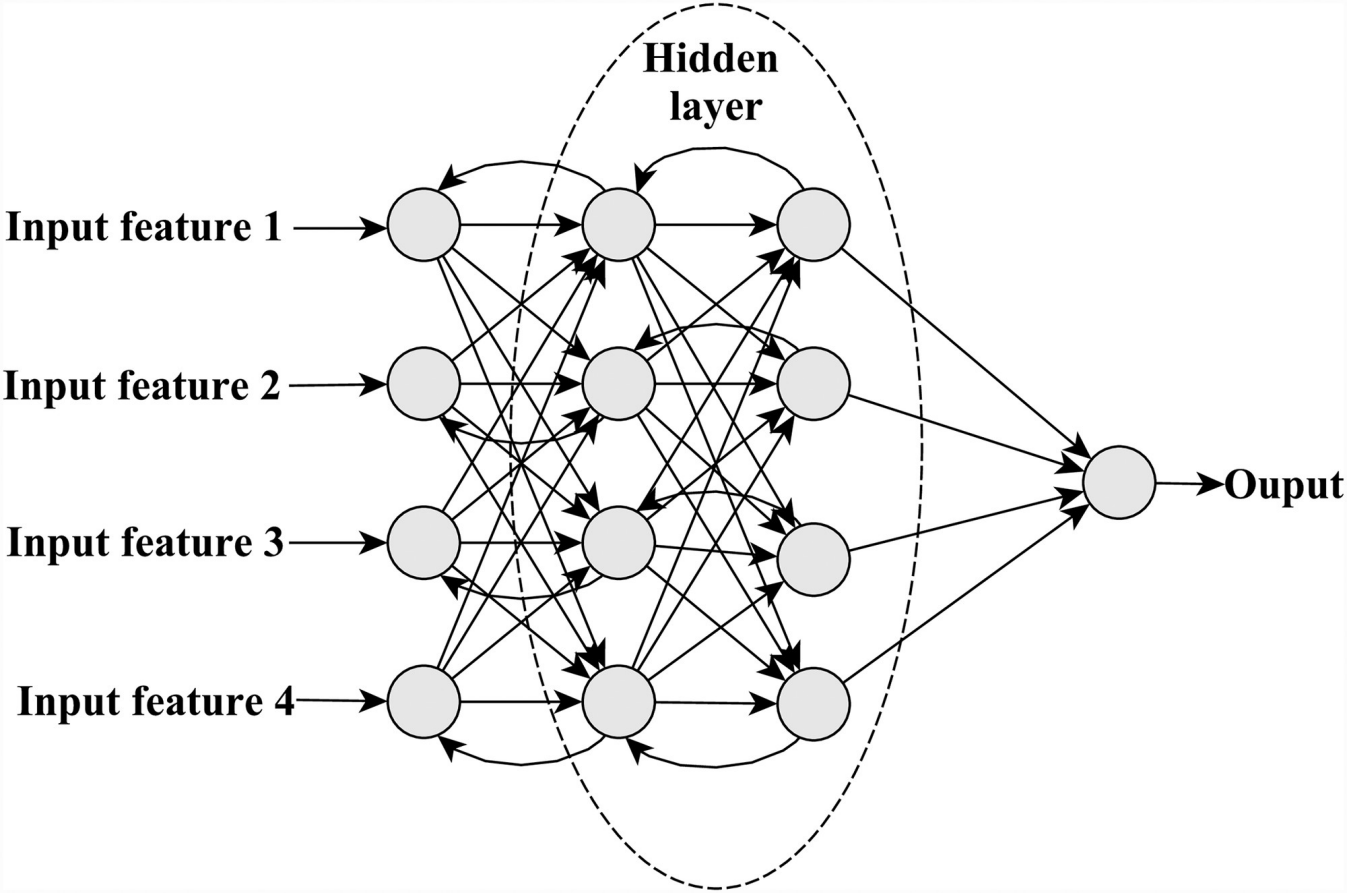
Neural network framework.

The proposed model has two hidden layers which have 100 neurons each. The input layer is fed with the weighted features (refer Section 3.3) which serve as input to the Neural Network. There is a single output layer which provides the strength of the relationship between the authors (real value) and hence the output layer has been designed with a single neuron. The number of neurons in the two hidden layers have been tuned in accordance with the training data, to provide the best result. Rectified Linear Unit activation function [37] was used. The optimizing algorithm that we have used for minimizing the objective function is the Adaptive Moment Estimation or in short, Adam’s optimizing algorithm [38] which is actually an extension of the stochastic gradient descent optimizer. While the latter maintains a single learning rate for all the weight updates, the Adam’s optimizer maintains a learning rate for each weight parameter which is separately adapted as the learning proceeds. Individual adaptive learning rates are computed for different parameters from the first and second moment estimates of the gradients. The algorithm is computationally very effective with low memory requirements and is suitable for problems with huge data and those with noisy or sparse gradients. The structure of the neural network is portrayed in Fig 3. The kernel initialization technique used is detailed in Section 6.1.

### 6.1 Modified kernel initializer

The kernel initializer initializes the weights to be used as input for each of the hidden units. Bhatia et al. [39] proposed a new weight initialization method for neural networks, instead of random initialization. According to them, weight initialization is a major factor that affects the speed of training a neural network. However, we cannot employ their method of weight initialization as their inputs are taken from a uniform distribution. The feature vectors given to our architecture follow a beta distribution. Hence, we have changed the kernel initializer to initialize the weights such that they follow the beta distribution. When the weights are initialized according to the distribution of the input features, convergence takes place faster and the learning is likewise better (refer to Table 10). Thus, weight initialization is vital to neural network learning because the choice of weights significantly impacts the quality of the neural network.

The Beta distribution is shaped by the parameters *α* and *β* and is defined by the function specified in Eq 10. In this equation, B refers to the Beta function.

$$\begin{array}{c} f\left(x;\alpha ,\beta \right)=\frac{1}{B(\alpha ,\beta )}x^{\alpha -1}{\left(1-x\right)}^{\beta -1} \end{array}$$ (10)

$$\begin{array}{c} Mean=\frac{\alpha }{\alpha +\beta }=\frac{3}{6}=0.5 \end{array}$$ (11)

$$\begin{array}{c} Var=\frac{\alpha \beta }{{\left(\alpha +\beta \right)}^{2}\left(\alpha +\beta +1\right)}=0.0357 \end{array}$$ (12)

*α* and *β* are the shape parameters, where *α*,*β*>0. The beta distribution is a discrete distribution. The values of *α* and *β* were both set to 3, which fetches the mean and variance given in Eqs 11 and 12.

## 7 Experiments

The link strength prediction framework was implemented in Python. The network was initially split into different time periods, based on the chosen time granularity. The weight of the links was computed based on the importance of each heterogeneous link, such as author-paper, author-topic and author-venue. Meta-path-based features were then computed for all time periods for the meta-path relations Author→Paper→Author, Author→Topic→Author and Author→Venue→Author. The future meta-path-based feature was forecast using the ARIMA model, based on the past and the current feature values. The neural network was used to predict the strengths of the links at a future time period. The link strength prediction experiments were carried out on two bibliographic datasets, details of which are provided in Sections 7.1 and 7.2.

### 7.1 The DBLP dataset

For the experiments, we used the DBLP dataset [40] (DBLP-Citation-network V3). A total of 1,86,952 papers, 1,93,189 authors, 25 topics and 634 venues for papers published between 1960 and 2011 were considered for our work. The following details were extracted: the year in which a paper was published, the paper’s author/s, the subject category the paper falls into and, finally, the conference in which it was published.

### 7.2 The HepTh citation network

The Arxiv HEP-TH is the High Energy Physics-Theory citation graph [41] and consists of 27,770 papers, 22,823 unique authors and 3,52,807 edges. This dataset holds all the papers for the 11-year period, 1992 to 2003. For link strength prediction, we have considered the author and paper nodes and the meta-path relation, author-paper-author.

### 7.3 Results and evaluation

For the dataset [40], a weighted network (Refer to Section 3.2) was constructed for a singular-year time granularity for each of the years between 1990 and 2009. The proposed weighted features for a specific meta-path relation, namely, the Weighted-Path-Count, Normalized-Weighted-Path-Count, Weighted-Activity-Ratio and Weighted-Symmetric-Activity-Ratio, were extracted (Refer to 3.3) for these time intervals for every author pair. The features were stored in a matrix form separately, and the matrices for all the time intervals were given as input to the ARIMA model to forecast the feature values for a future time interval. This procedure was repeated for all the features. Before using the ARIMA model, the time series was made stationary by using the log transformation, followed by the exponentially weighted moving average. The ARIMA model uses the parameters (1,0,0) for *p* (number of autoregressive terms), *d* (number of non-seasonal differences), and *q* (number of lagged forecast errors). Thus, only the autoregressive component of the ARIMA model was used as the fit was the best with the existing data. The ARIMA model produced feature values for the year 2010. These were given as input to the learning framework, which predicted the strength of the relationship between the authors for the specified meta-path relation. The framework was trained with the 2009 network, while the 2010 network was taken as the test data.

Along with link strength prediction, link prediction was also done by modifying the output layer of the neural network model. In the output layer, which is the last layer, instead of using linear activation (for the regression task), we use a sigmoid activation for the classification task which predicts the presence of a link (denoted by an output of 1) or its absence (denoted by an output of 0). The link prediction task was done using the test set. The training set contains links that were not connected in the past time periods. These links may remain disconnected in the future or may get connected. Whether a node pair will be connected in the future or not is learned by the link prediction model. The test set contains node pairs that are not connected in the current time period.

#### 7.3.1 Link strength prediction for the author-paper-author relation

Initially, the meta-path relation, author-paper-author, was considered, and the learning framework tested with the 2010 network. We consider only those authors who have published more than 20 papers for our link prediction task. There are about 2958 authors who have published more than 20 papers. There are a total number of 1,36,506 links in 2010 test set. The actual link strengths between the authors were computed from the dataset directly and taken as the ground truth. These actual weights were compared with those obtained through the learning model. The mean absolute percentage error (MAPE) was used to compare the results. The formula for the same is given in Eq 13, where AValue is the actual value and PValue the predicted value.

$$\begin{array}{c} MAPE=\frac{1}{n}\sum_{i=1}^{n}|\frac{AValue-PValue}{AValue}|*100 \end{array}$$ (13)

When these actual weights were compared with those obtained through the learning model, a mean absolute percentage error of 23% was obtained. The observed error value resulted from the erratic nature of certain authors who collaborated during their work together over a period of time, and subsequently went on to sever the connection altogether.

#### 7.3.2 Link strength prediction for the author-topic-author relation

Next, we considered the meta-path of the author-topic-author. This is a very special path where two authors are linked because of a shared common topic, without having had a paper published together either. The same procedure was carried out for this meta-path relation as well. When the actual weights were compared with those obtained through the learning model, a mean absolute percentage error of 38% was obtained. The reason for this error value can be attributed to the varying topics that the two authors might be interested in, which is impossible for the learning algorithm to capture, as the meta-path relation only computes the strength based on the entirety of the topics the two authors have written on. Moreover, the meta-path relation A-T-A has the least importance in predicting future links when compared with the other two meta-path relations.

#### 7.3.3 Link strength prediction for the author-venue-author relation

The same experiment was repeated with the author-venue-author meta-path, which yielded a mean absolute percentage error of 29%. The venue nodes between two authors are not as varied as the topic nodes between them, resulting in a lower error value when compared with that of the author-topic-author relation. Two authors connected by a venue have greater chances of publishing a paper together than when connected by topic, because their geographic location plays a pivotal role in identifying future co-authors when compared with the A-T-A relation.

The results of the classification and regression for link and strength predictions are recorded in Table 1 for all the three meta-path relations.

**Table 1:** Performance of different meta path relations.

Meta-Path	Link prediction accuracy	MAPE for link strength prediction
A-P-A	88.61%	23%
A-T-A	62.31%	38%
A-V-A	75.88%	29%

#### 7.3.4 Link strength prediction for HepTh dataset

For the Hep-Th dataset [41], a singular-year time granularity was chosen for the period 1992 to 2002. The author-paper-author meta-path relation was considered. The actual link strengths were computed for the year 2003 and taken as the ground truth against which the results of the learning framework were compared. It yielded a mean absolute percentage error of 27.07% for link strength prediction and an accuracy of 87.16% for link prediction.

The first evaluation was carried out to analyze the effects of the varying time granularity described in Sections 7.3.5 and 7.3.6.

#### 7.3.5 Setup for varying time granularity

Initially, the network was sliced into subnetworks at regular time intervals, and papers published between 1960 and 1995 were considered. The first time granularity that was taken up was 4 years. The same network was constructed for a 3-year time granularity, followed by a 2-year time granularity. Details of the total number of papers, published during the different time slices corresponding to the 4-year time interval, are given in Table 2. Details of those published during the different time slices corresponding to the 3-year time interval are provided in Table 3 and papers published during different time intervals corresponding to the 2-year time period are shown in Table 4.

**Table 2:** Papers published in 4-year time interval.

Period	Published papers
1960-1963	16
1964-1967	55
1968-1971	130
1972-1975	457
1976-1979	808
1980-1983	1678
1984-1987	3271
1988-1991	7089
1992-1995	12597

**Table 3:** Papers published in 3-year time interval.

Period	Published papers	Period	Published papers
1960-1962	13	1978-1980	714
1963-1965	25	1981-1983	1367
1966-1968	81	1984-1986	2144
1969-1971	82	1987-1989	3707
1972-1974	289	1990-1992	6938
1975-1977	573	1993-1995	10168

**Table 4:** Papers published in 2-year time interval.

Period	Published papers	Period	Published papers
1960-1961	8	1978-1979	403
1962-1963	8	1980-1981	708
1964-1965	22	1982-1983	970
1966-1967	33	1984-1985	1253
1968-1969	69	1986-1987	2018
1970-1971	61	1988-1989	2580
1972-1973	88	1990-1991	4509
1974-1975	369	1992-1993	5519
1976-1977	405	1994-1995	7078

#### 7.3.6 Link strength prediction for varying time granularities

The experiment for varying time granularities was conducted using the DBLP dataset for the author-paper-author relation. For the 4-year time granularity, the data (meta-path features) from 1960 to 1995 was split into 4-year time intervals and given as input to the ARIMA model, which provided the feature values for the interval 1996 to 1999. The values were given as input to the neural network. The actual papers published between 1996 and 1999 were taken as the test data. The weighted network was constructed for the test data with the actual weights obtained from the dataset, which was taken as the ground truth. These actual weights were compared with those obtained through the neural network learning model. The mean absolute percentage error for the test data was 26.19%. The same procedure was repeated for the 3-year time granularity, with the data from 1960 to 1995 split into three-year time slices and given as input to the ARIMA model. The forecast feature values were obtained for the period 1996 to 1998 and the ground truth was obtained from the dataset for the same. When the output of the learning model was compared with the ground truth, the MAPE was found to be 24.56%. For the two-year time granularity, the data from 1960 to 1995 was given as input to the ARIMA model. A forecast was made for the features for the years 1996-1997 and the ground truth was constructed for the same from the dataset. When the feature values obtained from the ARIMA model were given as input to the neural network learning model and its output compared with the ground truth, the MAPE was found to be 19.02%. The results are depicted in Table 5.

**Table 5:** Mean Absolute Percentage Error(MAPE) for different granularities of time.

Time Granularity	MAPE
4-year period	26.19%
3-year period	24.56%
2-year period	19.02%

The error values obtained represent the unpredictable nature of authors who work together as random associates for a while because of sheer necessity and, in due course, terminate the connection altogether.

When the time interval decreases, the number of inputs given to the ARIMA model increases. The 2-year time interval provides more inputs to the ARIMA model (past values) and hence provides greater accuracy to the output of the ARIMA model among the time granularities considered. This, in turn, increases the accuracy of the predicted strength values between the authors. Thus, considering past time intervals of a finer time granularity results in an improved link strength prediction performance between nodes.

#### 7.3.7 Link strength analysis between author pairs

**Fig 4:**
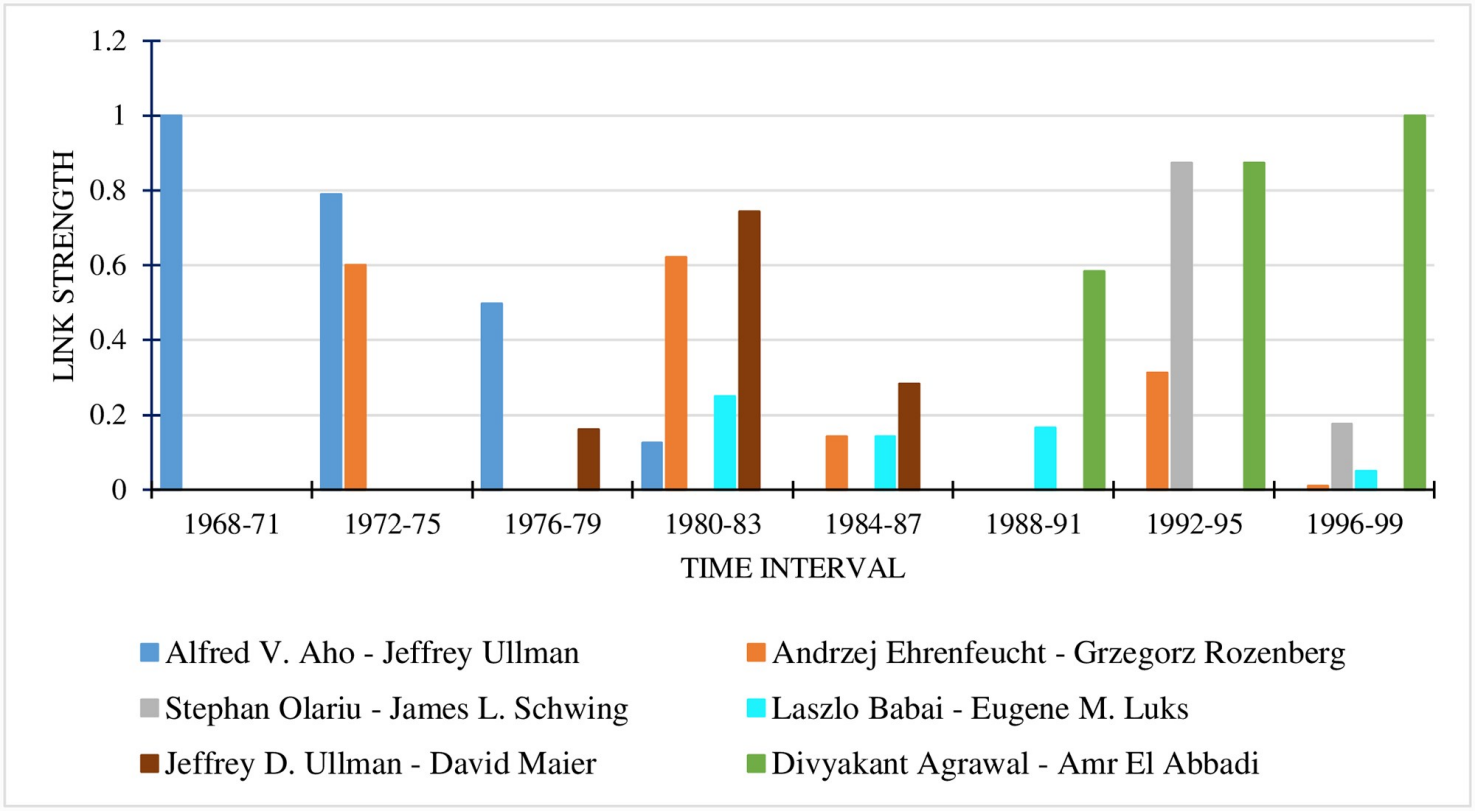
Link strengths between authors for 4-year time interval.

**Fig 5:**
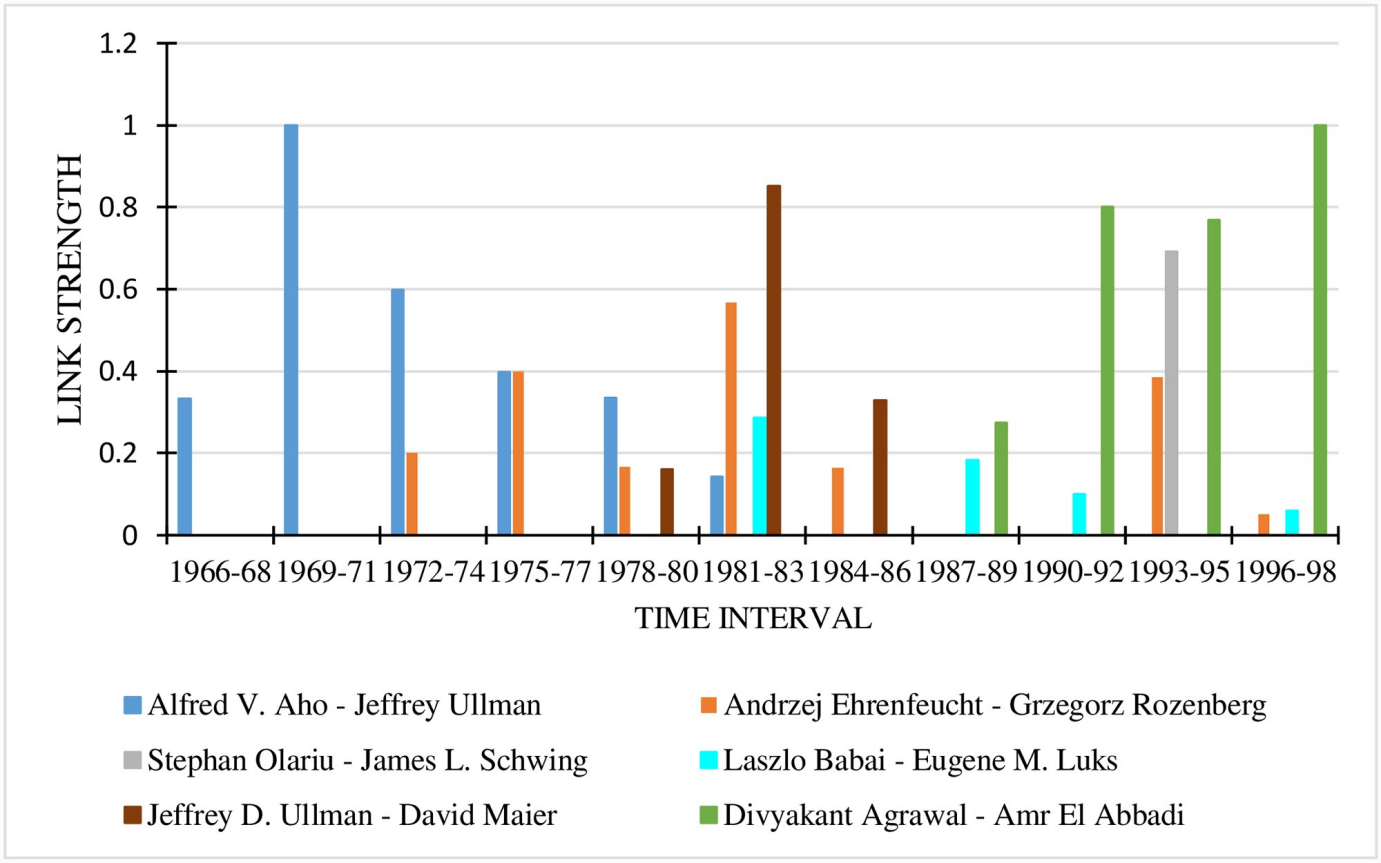
Link strengths between authors for 3-year time interval.

**Fig 6:**
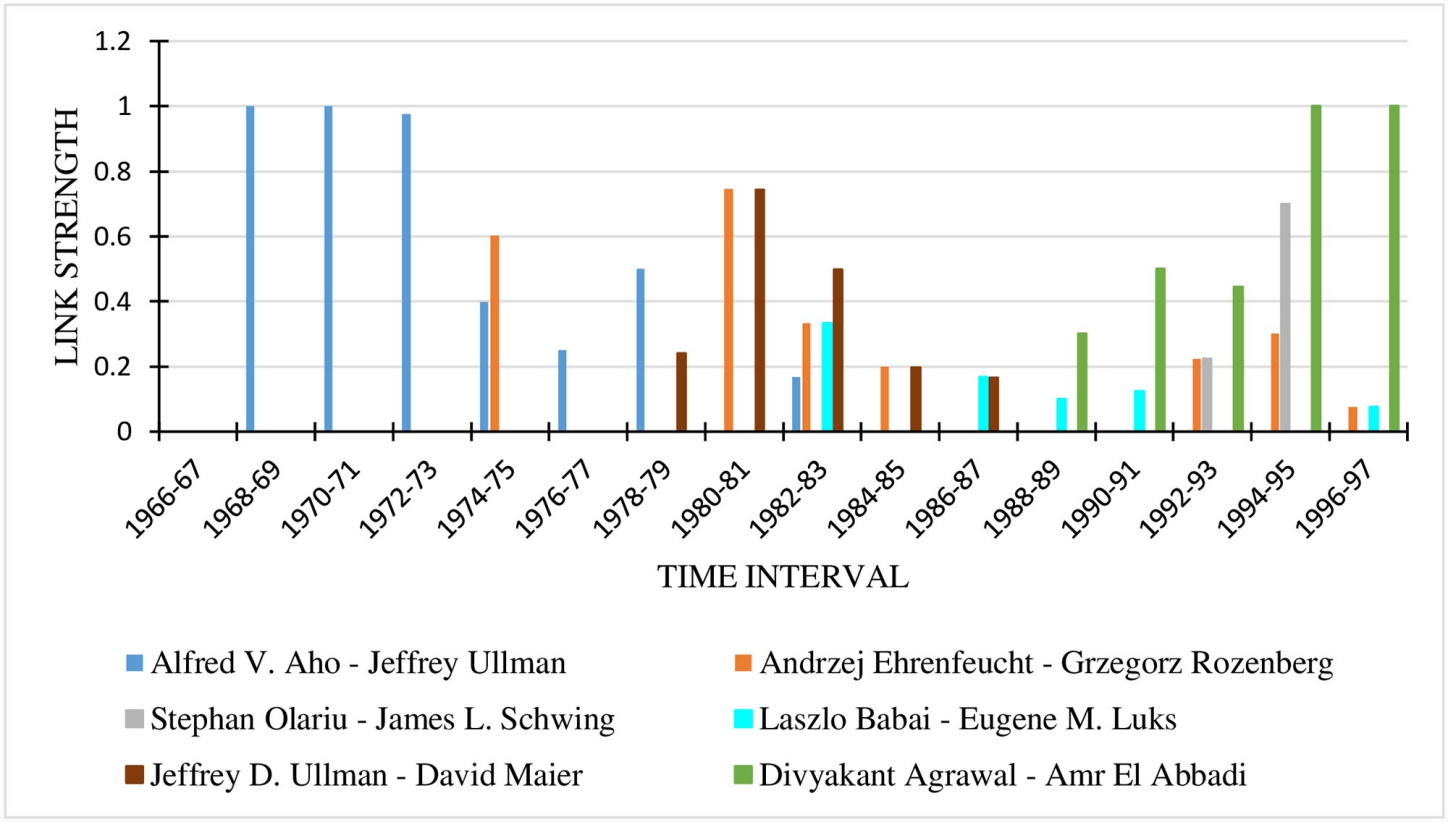
Link strengths between authors for 2-year time interval.

The relationship strength between 10 author pairs was drawn for 4-year, 3-year and 2-year time granularities. The details are provided in Figs 4, 5 and 6 respectively. In all the graphs, the last time interval shows the predicted values of the link strength.

For the 4-year time interval (Fig 4), the most popular author pair for the period 1996-1999 is Divyakant Agrawal and Amr El Abbadi, while those for the time period 1992-1995 are Divyakant Agrawal—Amr El Abbadi and Stephan Olariu—James L. Schwing. Considering the 3-year time interval (refer to Fig 5), the most popular author-pair for the period 1996-1998 is Divyakant Agrawal—Amr El Abbadi. It is seen that the author pair Stephan Olariu—James L. Schwing is absent in the interval 1996 to 1998, meaning that their link strength is 0, indicating that they did not collaborate then. A comparison of the two figures clearly indicates that the two authors collaborated in 1999. The fact that the Stephan Olariu—James L. Schwing pair did not collaborate during the years 1996-1997 is proved by Fig 6.

When a finer time granularity is taken, accuracy increases. The graphs make it plain that when we consider a time granularity of a higher duration, we miss such intricate details of the relationship strength like the time at which it changed.

#### 7.3.8 Link strength prediction in various heterogeneous networks

**Fig 7:**
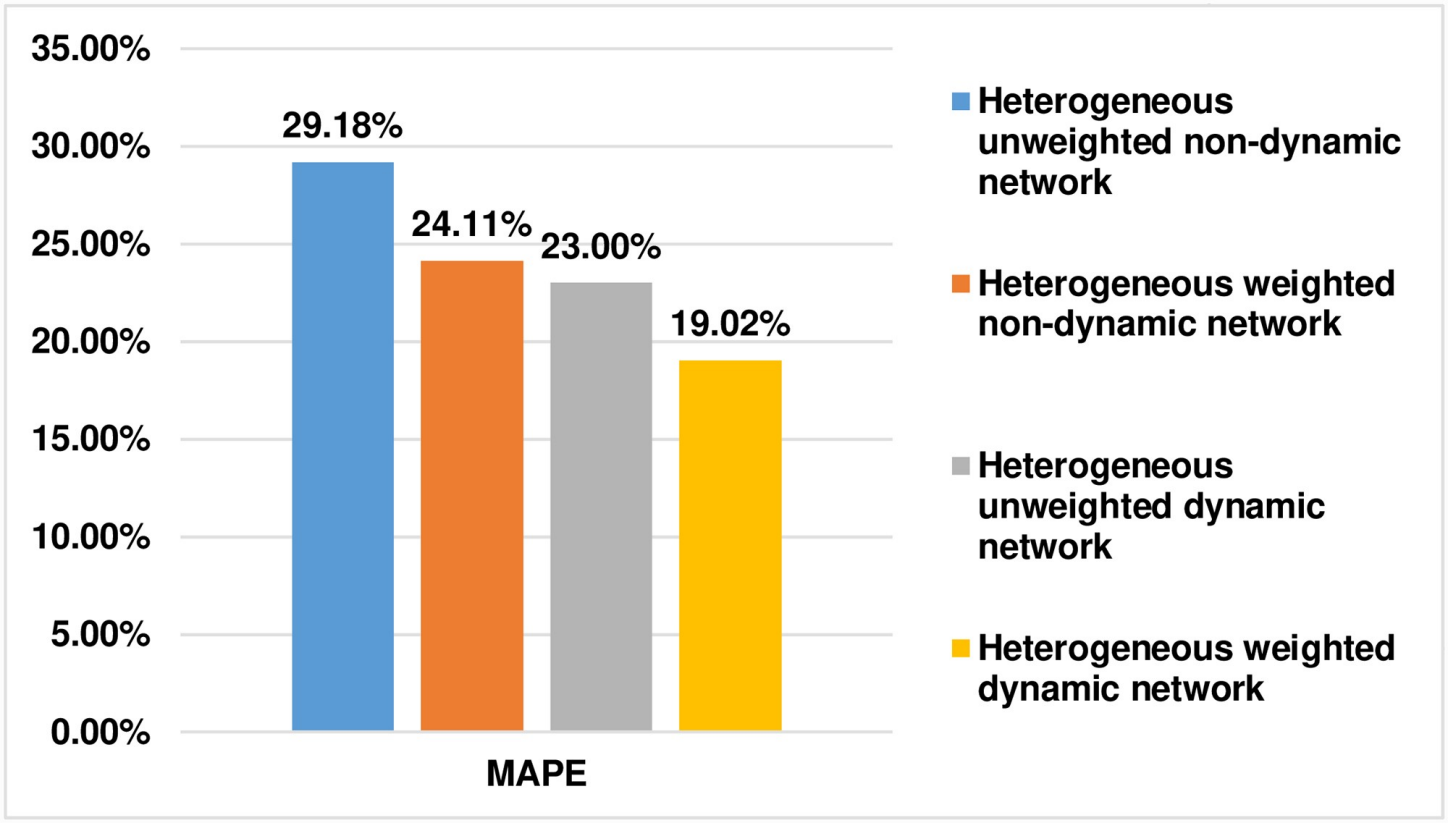
MAPE for various types of heterogeneous networks.

The link strength prediction model proposed for the heterogeneous weighted dynamic network was evaluated against other types of networks such as the heterogeneous unweighted non-dynamic network, the heterogeneous weighted non-dynamic network, and the heterogeneous unweighted dynamic network. For this evaluation, we constructed a heterogeneous network using the DBLP dataset [40] for the author-paper-author relation. For dynamism, we used a 2-year time granularity. The results obtained are shown in Fig 7.

From this result, it can be inferred that both link weights and dynamism play a pivotal role in link strength prediction.

#### 7.3.9 ARIMA vs other forecasting models

The experiments for ARIMA, Bayes and LSTM forecasting were conducted using the DBLP dataset for the author-paper-author relation. For the 4-year time granularity, the data (meta-path features) from 1960 to 1995 was split into 4-year time intervals and given as input to the forecasting models independently, which provided the feature values for the interval 1996 to 1999. The forecast feature values were given as input to the neural network. The actual papers published between 1996 and 1999 were taken as the test data. The weighted network was constructed for the test data with the actual weights obtained from the dataset, which was taken as the ground truth. These actual weights were compared with those obtained through the neural network learning model. The results using ARIMA, Bayes and LSTM forecasting models are specified in Table 6.

**Table 6:** Comparison of ARIMA with other forecasting models.

MAPE for ARIMA forecasting	MAPE for Bayes forecasting	MAPE for LSTM forecasting
26.19%	29.58%	24.69%

#### 7.3.10 Usage of multiple weighted features

Yang and Yang [28] proposed a single weighted feature for link prediction in a weighted heterogeneous network. We compared our work using 4 weighted features (refer to Section 3.3) against a single weighted feature, namely, the Weighted Path Count for the meta-path relation, author-paper-author, for the 2-year time interval. Except for the use of multiple features, the other setup remained the same and the experiment was carried out using the DBLP dataset [40]. The mean absolute percentage error obtained for the experiment using a single feature was 28.47%, as opposed to 19.02% when done for 4 features. The experiment was also tried with 2 and 3 features and the results are illustrated in Table 7.

**Table 7:** Performance due to the usage of multiple weighted features.

Weighted Features	MAPE
WPC	28.47%
WPC+NWPC	26.56%
WPC+NWPC+WAR	22.26%
WPC+NWPC+WAR+WSAR	19.02%

Table 7 depicts that the use of 4 features for the proposed link strength prediction model results in greater accuracy than the use of fewer features.

#### 7.3.11 A comparison of our work with state-of-the-art methods

We have compared our work (*NN*–*Framework*) with certain methods that have been known to produce good results for link prediction. We used the A-P-A relation with all the four weighted features for the time period between 1990 and 2009, using a one-year time interval for link prediction. The dataset we used to test this was the DBLP dataset [40]. The first method that we used to test against our model was the Common Neighbors without weights in a static homogeneous network. Then, a heterogeneous network was constructed using the above-said dataset with a single weighted feature [28], and the method referred to as YangMining. Link prediction was done using the Naive Bayes classifier, as in the paper. In this method, the data from 1990 to 2009 was taken for training and the 2010 data for testing. Further experimentation was carried out with the Common Neighbors algorithm by constructing a weighted homogeneous network using the ARIMA model [32], where the data spanning the years 1990 to 2009 was used as input to the ARIMA model with a single-year time granularity. The forecast for the Common Neighbors was done for the year 2010, the results tabulated in Table 8 and referred to as *CN*–*TimeSeries*. Finally, with the same dataset, the PathPredict [35] was also implemented. The link prediction results in terms of accuracy are tabulated in Table 8.

**Table 8:** Comparison of link prediction results with state-of-the-art techniques.

Method	Accuracy
Common Neighbours	60.39%
YangMining	68.32%
*CN*–*TimeSeries*	71.83%
PathPredict	73.54%
*NN*–*Framework*	88.61%

#### 7.3.12 A comparison of the neural network with other regression algorithms

Link strength prediction for the 2-year time granularity for the author-paper-author meta-path relation was done with other regression algorithms using the dataset [40], and the results compared with that of the learning strategy proposed. The data for the years 1960 to 1997 was used for the experiment, and the results recorded in Table 9. It can be seen that the proposed model yielded better results than other algorithms, demonstrating that neural nets can be used in applications where traditional algorithms fail. The reason can be attributed to the multiple layers in neural networks that simulate the functioning of the brain. Also, the self-learning nature of neural nets ensures that the results are much more accurate.

**Table 9:** Mean Absolute Percentage Error(MAPE) for different regression algorithms.

Algorithm	MAPE
Deep Neural Network	19.02%
Linear Regression	55.40%
Lasso Regression	37.82%

#### 7.3.13 A performance analysis of the beta kernel initializer

A weighted heterogeneous network was constructed for the meta-path relation, author-paper-author, for the 2-year time interval using the dataset [40] for the years 1960 to 1997. The link strength prediction experiment was done using the RandomUniform kernel initializer, RandomNormal, as well as the proposed beta kernel initializer. As expected, the beta kernel initializer fetched a lesser mean absolute percentage error, as shown in Table 10. Moreover, while the RandomUniform kernel initializer achieved convergence in 1000 epochs, the proposed beta kernel initializer was able to achieve a lesser MAPE value in 850 epochs. The RandomNormal kernel initializer achieved a MAPE value greater than that obtained using the RandomUniform one in 1000 epochs.

**Table 10:** Performance of different kernel initializers.

Kernel Initializer	Epochs	MAPE
Random Uniform	1000	22.56%
Beta Kernel	850	19.02%
Random Normal	1000	23.01%

## 8 Conclusion and future work

In this paper, a link strength prediction framework was proposed for a weighted dynamic heterogeneous network. Initially, weighted networks were constructed for regular time intervals and weighted meta-path-based features extracted for each of these time intervals. This was followed by the use of the ARIMA forecasting model to predict the weighted features for a future time period, based on the past and current values given to it as input. These future feature values were given as input to the proposed supervised neural network learning algorithm which predicts the weights of links between authors in a future time period. The experiment was repeated for different granularities of time, namely, 4-year, 3-year and 2-year. It was observed that the 2-year time granularity yielded link strength values with a smaller error percentage. Besides, the experiment was repeated for different meta-path relations like the author-topic-author and author-venue-author. Initially done for the author-paper-author relation, it was observed that this meta-path achieved a higher percentage of accuracy in terms of link strength values between authors. The proposed method outperforms other algorithms by yielding a MAPE value which is far less than that of other algorithms. Moreover, considering a weighted dynamic heterogeneous network yields very good results when compared to other networks like the heterogeneous unweighted non-dynamic network, the heterogeneous weighted non-dynamic network, and the heterogeneous unweighted dynamic network. Likewise, using the beta kernel initializer has enhanced the performance of the proposed model. However, our framework has a gap. Our method is distinctive in predicting future link strengths between authors. However, apart from the existing nodes (which may be, for instance, of the type authors/topics/venues), the probability of new nodes cropping up in due course in a future time interval has not been captured by our framework, which restricts itself to predicting future links and their strengths. In future research, we intend to consider the evolution of new nodes as well, thereby predicting their occurrence at a future interval in time. We would also like to experiment with Graph Neural Networks as a part of our future research.
